# Effect of pilose antler polypeptide on the mechanism of bone homeostasis in osteoporosis

**DOI:** 10.3389/fmed.2023.1289843

**Published:** 2023-11-01

**Authors:** Guochen Wang, Yubo Meng, Wensi Ouyang, Changwei Zhao, Wenhai Zhao

**Affiliations:** ^1^Changchun University of Chinese Medicine, Changchun, China; ^2^College of Traditonal Chinese Medicine, Changchun University of Chinese Medicine, Changchun, China; ^3^The Affiliated Hospital to Changchun University of Chinese Medicine, Changchun, China

**Keywords:** osteoporosis, bone homeostasis, pilose antler polypeptide, osteoblasts, osteoclast

## Abstract

Osteoporosis stands out as a prevalent metabolic disorder, bearing significant repercussions on human well-being and overall quality of life. It remains an urgent concern within the global public health framework due to its widespread occurrence. Osteoporosis arises from an abnormal metabolism in osteoblasts and osteoclasts, resulting in a disruption of the delicate equilibrium between bone formation and bone resorption. Within this context, deer antler peptides emerge as natural active compounds, wielding a pivotal role in governing the differentiation, proliferation, and mineralization of osteoblasts, as well as influencing the activity of osteoclasts. This article aims to consolidate our comprehension of the mechanisms underpinning the dynamic balance between bone formation and resorption, meticulously orchestrated by osteoblasts and osteoclasts in osteoporosis. Furthermore, it offers a comprehensive overview of how deer antler peptides, through their modulation of relevant signaling pathways, contribute to the enhancement of bone homeostasis. These insights deepen our understanding of the pathological processes through which deer antler peptides ameliorate bone homeostasis, while also presenting novel strategies for osteoporosis management.

## Introduction

Osteoporosis (OP) manifests as a condition marked by a reduction in bone density, resulting in heightened bone fragility and vulnerability to fractures (1). It is reported by the National Osteoporosis Foundation in the United States, the persistent aging of the global populace is poised to trigger a substantial surge in osteoporosis cases (2). Projections indicate that by 2030, the global tally of adults grappling with osteoporosis and diminished bone density will surpass 200 million (2). Moreover, a daunting economic impact anticipated in the United States by 2040, the financial strain attributed to osteoporosis-linked fractures is estimated to soar to an astounding 50 billion USD, presenting a formidable challenge to public health (2, 3).

The fundamental pathological mechanism underlying osteoporosis (OP) is an aberration in bone metabolism and disruption of bone homeostasis, notably characterized by a substantial reduction in bone formation capacity and heightened bone resorption (3). At the cellular level, this manifests as a diminution in the expression of osteoblasts (OBs) and an escalation in osteoclasts’ (OCs) activity (4). Ultimately, these alterations culminate in a thinning of cortical bone, a decrease in trabecular numbers, and an increase in trabecular spacing, collectively rendering the bones fragile (4). Therefore, upholding a dynamic equilibrium between OBs and OCs emerges as pivotal for both the structure and development of bones.

Pilose antler polypeptide/velvet antler polypeptide (PAP/VAP), a potent extract derived from traditional Chinese medicine, deer antler, effectively promotes the proliferation of osteoblasts (OBs) and enhances bone cell mineralization ability (5), which amplifies the expression and activity of essential factors like BMP-2, ALP, estrogen, and bone-protective agents, consequently augmenting calcium and phosphorus levels within the human body (5). Moreover, it demonstrates the capability to suppress inflammatory factors and osteoclast activity. By multiple pathways such as MAKP, EGF, NF-κB, BMP-2, insulin, ERK, and PI3K/Akt, PAP significantly contributes to maintaining a balanced bone homeostasis (5). So the article commencing with an exploration of the pathological mechanisms of osteoporosis (OP) comprehensively outlines the pertinent effects of PAP on bone metabolism, offering fresh insights into the mechanisms by which PAP intervenes in OP.

## Dynamic balance between osteoblasts and osteoclasts

At present, bone mineral density remains the principal diagnostic parameter for osteoporosis (OP) employing dual-energy X-ray absorptiometry (DXA) to calculate the T-score. A T-score of <−2.5 is indicative of osteoporosis (3). This diagnostic approach stems from the primary pathological feature of OP, which is the decline in bone mass. Bone mineral density assessment offers valuable insights into the abundance of bone mass within the human body (6). The quantity of bone mass is contingent on the population of bone cells, while an aspect intimately tied to the activity of osteoblasts (OBs) and osteoclasts (OCs) (7). Thus, maintaining a dynamic equilibrium between OBs and OCs emerges as pivotal in the onset and progression of OP.

Osteoblasts (OBs) represent a subset of undifferentiated monocytes originating from mesenchymal stem cells (MSCs) within the bone marrow. The differentiation of mesenchymal stem cells into osteoblasts predominantly hinges on signaling pathways such as BMP (bone morphogenetic protein) and Wnt/β-catenin, although pathways like NF-κB also play a contributory role (8). The differentiation sequence initiates as mesenchymal stem cells undergo transformation into osteo-chondroprogenitor cells. These cells, when activated by osteogenic transcription factors like runt-related transcription factor 2 (Runx2), drosophila distal less 5 (DLX5), and osterix (OSX), subsequently progress into pre-osteoblasts (9). Early osteogenic genes, inclusive of bone-derived alkaline phosphatase (BALP) and collagen1ɑ1 (COL1A1), along with typical osteoblast markers like bone sialoprotein II (BSP II), osteocalcin (OCN), and osteopontin (OPN), govern the transcriptional pathway guiding the maturation of pre-osteoblasts into fully mature osteoblasts (8, 9).

During the proliferation phase of osteoblasts, notable alterations take place within the Golgi apparatus and endoplasmic reticulum of their organelles (10). Vesicle transport becomes highly coordinated at the plasma membrane, facilitating close interactions between neighboring osteoblasts (11). As osteoblasts progress towards differentiation, they initiate the production of bone matrix within their cells. This matrix encompasses adjacent osteoblasts, progressively giving rise to bone tissue (12). Subsequently, through the deposition of hydroxyapatite calcium, the bone tissue undergoes mineralization alongside the extracellular matrix, ultimately contributing to the development of human bones (10–12).

A healthy skeleton does not maintain continuous generation and necessitates the bone resorption process, in which osteoclasts (OCs) play a vital role in clearing aged and damaged bone. OCs are multinucleated cells formed by the fusion of mononuclear precursor cells and are considered terminally differentiated cells. These cells originate from mononuclear hematopoietic myeloid lineage cells, where myeloid progenitor cells transform into pre-monocytes under the stimulation of PU.1 and MITF (13). Subsequently, under the influence of M-CSF, pre-monocytes progress into osteoclast precursors (13). Various cell types, including bone marrow stromal cells, T cells, osteoblasts, and B cells, have the capacity to upregulate the expression of receptor activator of nuclear factor-kappa B ligand (RANKL), which serves as an NF-κB ligand receptor (14). Then RANKL activates the RANK receptor on osteoclasts (OCs) initiating a cascade that transforms osteoclast precursors into osteoclasts (14). This activation stimulates the proliferation, differentiation, and multinucleation of osteoclasts (14). Mature osteoclasts manifest as multinucleated cells with adhesive molecules and a dynamic cell skeleton. On the other hand, osteoblasts can secrete acid proteases and modify the microenvironment of adhesion sites, leading to a reduction in collagen within bones and the breakdown of aged and damaged bone tissue (15, 16). Therefore, the harmonious coordination and equilibrium between osteoblasts and osteoclasts are pivotal, ensuring normal bone growth and representing critical factors in maintaining bone mass within the human body.

Influenced by factors such as aging, hormone levels, and other diseases, the expression levels of osteoblasts (OBs) and osteoclasts (OCs) experience shifts. This disruption upsets the delicate balance between them, culminating in suppressed bone formation and heightened bone turnover and resorption. As a result, there is an imbalance in bone homeostasis, weakening not only the material properties of bone, such as mineral size and collagen, but also triggering adverse alterations in bone shape and structure. These changes include a decrease in trabecular thickness, alterations in connectivity, reduction in cortical bone thickness, and enlargement of pores (17, 18). Ultimately, these alterations contribute to the pathological structure characteristic of osteoporosis.

As individuals age, a plethora of aging factors manifest within the body, encompassing DNA damage, heightened oxidative stress, telomere shortening, and chromatin deformation, among others. These factors not only diminish the differentiation capacity of bone marrow mesenchymal stem cells into osteoblasts but also impair their functionality and regenerative potential (19, 20). Moreover, they result in a reduced expression of crucial transcription factors like RUNX2, osterix, and nuclear factor erythroid 2-related factor 2 (Nrf2), thereby exacerbating bone resorption and disrupting bone homeostasis (21, 22).

The decline in estrogen levels is recognized as a significant contributor to this imbalance in bone homeostasis. Postmenopausal women, experiencing reduced estrogen levels, undergo a shorter lifespan of osteoblasts (OBs) and a relatively prolonged lifespan of osteoclasts (OCs) (23, 24). This discrepancy leads to diminished bone formation compared to bone resorption, ultimately resulting in an imbalance in bone homeostasis. Concurrently, an increase in RANKL expression can also provoke this imbalance. Research indicates that reduced estrogen levels can decrease osteoprotegerin (an antagonist to RANKL) and encourage elevated RANKL expression; RANKL, in turn, activates osteoclasts (OCs), heightening bone resorption rates (25, 26). Additionally, certain malignant bone diseases or immune disorders such as multiple myeloma and rheumatoid arthritis, or hormonal suppression in patients (e.g., females with breast cancer, males with prostate cancer), can augment the expression of RANKL (27–29). This heightened osteoclast activity contributes to an imbalance in bone homeostasis (4).

Inhibiting the principal pathways by which mesenchymal stem cells differentiate into osteoblasts is also a pivotal factor in the disruption of bone homeostasis. Research has uncovered abnormal expression of Forkhead Box F2 (FOXF2) in postmenopausal women with low bone mass; FOXF2 can hinder osteoblast formation via the Wnt 2b/β-catenin signaling pathway. Notably, when FOXF2 was knocked out in mice, an increase in bone mass was observed (30).

In specific cases, patients with osteoporosis (OP) and concurrent degenerative or hematological disorders may accumulate an excess of iron in their bodies. Excess iron can impede the Wnt signaling pathway, inducing morphological changes such as mitochondrial membrane shrinkage, condensation, loss, and outer membrane rupture. Additionally, this excess iron generates lipid peroxidation products (LPO) and reactive oxygen species (ROS), disrupting the differentiation process of osteoblasts (31).

## Pilose antler polypeptide/velvet antler polypeptide

As a prominent component of traditional Chinese medicine, Deer antler is sourced from the antlers of young, non-calcified, velvet-covered male sika deer or red deer, and it encompasses various active substances and amino acids (32). One of its significant constituents is PAP, a short peptide comprising several amino acids including glycine, glutamic acid, leucine, valine, and alanine (33). PAP, a natural active component extracted from deer antler, accounts for 50 to 60% of the total wet weight of deer antler (34). PAP plays a pivotal role in enhancing bone metabolism and addressing the imbalance in bone homeostasis (33–35).

**Figure 1:**
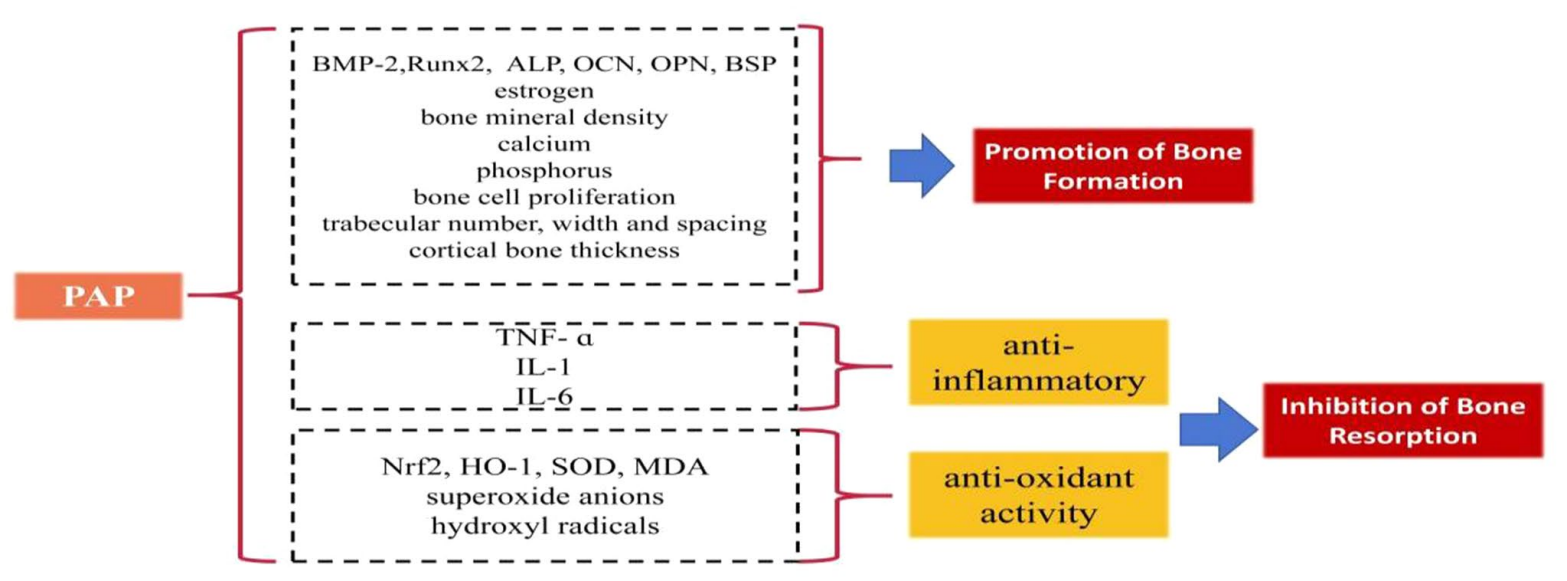
The beneficial role of PAP in regulating bone formation and resorption.

Some studies have elucidated the beneficial role of PAP in regulating bone formation and resorption (Figure 1):

### Promotion of bone formation

PAP has demonstrated significant potential in augmenting bone formation. In ovariectomized female rats, PAP administration led to increased levels of estrogen, BMP-2, and ALP, effectively promoting bone formation (36). Notably, when compared to the estradiol group (estradiol being a drug utilized to counter osteoporosis stemming from diminishing estrogen levels), PAP exhibited superior efficacy in enhancing bone mineral density (36). Moreover, when co-cultured with human osteosarcoma cells in isolated and purified forms, (OS-732), PAP displayed the ability to enhance ALP expression, further encouraging bone formation (37). *In vitro* culture studies involving rat cells revealed that varying concentrations of PAP resulted in increased BMP-2 levels, with the 400 μg/mL concentration of PAP demonstrating the most notable impact (38). Further *in vitro* cell culture investigations indicated that PAP augments the expression of the key transcription factor Runx2, crucial for osteogenesis. It also enhances the content of OCN, ALP, OPN, and BSP, thereby promoting bone formation (39). Long-term gavage administration of PAP in a rat model of ovariectomy-induced osteoporosis exhibited notable benefits. It elevated estrogen and ALP levels, and improved calcium and phosphorus levels, as well as enhanced bone mineral density (40, 41). PAP was also shown to increase bone calcium content in a rat model of retinoic acid-induced osteoporosis, effectively improving the quantity, width, and spacing of bone trabeculae. Additionally, PAP significantly stimulated bone cell proliferation, enhancing bone mass, density, and formation more effectively than calcium gluconate (42). Moreover, polylactide glycolide (PLGA) microspheres loaded with PAP demonstrated enhanced efficacy by not only improving trabecular area and cortical average thickness in ovariectomy-induced osteoporotic rats but also by enhancing the bioavailability of PAP, addressing the challenge of PAP’s short half-life (43).

### Inhibition of bone resorption

PAP effectively inhibits bone resorption through its anti-inflammatory and antioxidative effects. PAP displays the capability to diminish bone resorption by mitigating inflammation, which accomplishes this by reducing the levels of pro-inflammatory cytokines such as tumor necrosis factor-alpha (TNF-α), interleukin-1 (IL-1), and interleukin-6 (IL-6), (44–46). By doing so, it curtails the inflammatory response, safeguarding osteoblasts, and thwarting the induction of bone resorption prompted by pro-inflammatory cytokines (44–46). Furthermore, PAP exerts an antioxidative effect that contributes to the inhibition of bone resorption. It induces the production of superoxide dismutase (SOD), nuclear factor erythroid 2-related factor 2 (Nrf2), and heme oxygenase-1 (HO-1), while inhibiting malondialdehyde (MDA). This orchestrated response combats oxidative reactions, diminishing the proliferation of osteoclasts stimulated by oxidative factors and subsequently reducing the rate of bone resorption (47, 48). Additionally, enzymatic digestion and further purification of PAP yield a component of PAPs that exhibits highly potent antioxidant activity. This component showcases significant abilities in scavenging superoxide anions and hydroxyl radicals (49).

In addition to the mentioned effects, PAP holds promising potential in improving bone homeostasis by enhancing the immune system. Several *in vitro* experiments conducted on mice have shown that PAP can elevate the number of CD4(+)/CD8(+) cell subsets, enhance the cytotoxicity of NK cells, and boost the overall immune response in mice (50, 51). The immune system plays a significant role in bone resorption, as evidenced by multiple studies. The activation of the immune system often requires calcium and phosphate obtained from bone absorption induced by inflammatory factors (52–54). However, it’s important to note that while the potential link between PAP and immune system modulation for maintaining bone homeostasis is intriguing, there’s currently no confirmed animal research in this area, leaving it as an uncharted field. Further investigations are warranted to fully understand and validate this aspect.

## Signal pathway of pilose antler polypeptide improving bone homeostasis

### BMP-2/Smad1, 5/Runx2 signaling pathway

**Figure 2:**
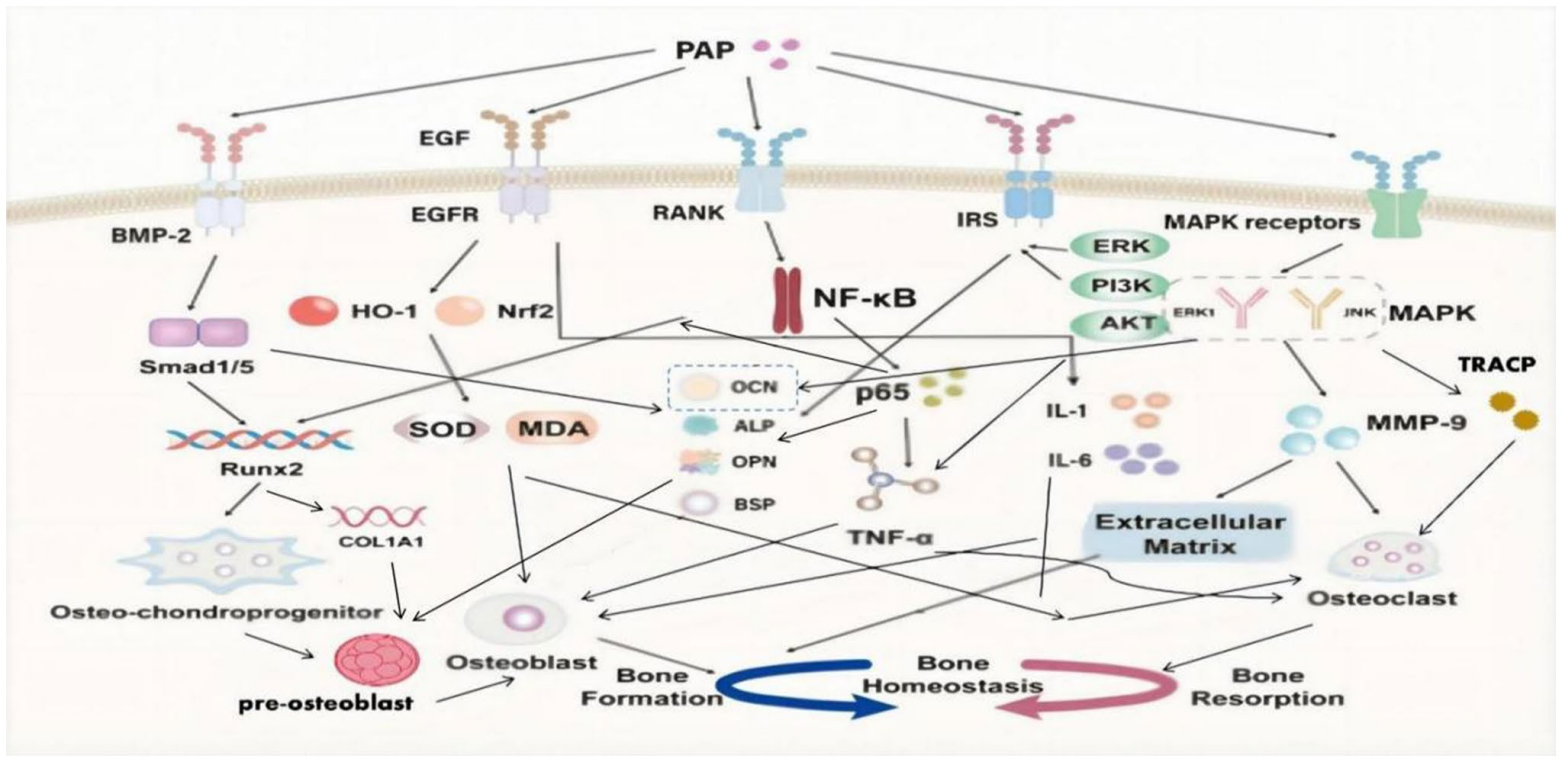
Signal pathway of deer antler peptides improving bone homeostasis.

Bone morphogenetic protein-2 (BMP-2) stands as a pivotal member within the BMP family, widely acclaimed for its robust osteoinductive properties. BMP-2 plays several critical roles in bone regeneration and repair (55, 56):

1. Recruitment and angiogenesis: BMP-2 plays a crucial role in enhancing the recruitment of osteochondral progenitor cells to specific bone formation sites. Additionally, it stimulates angiogenesis, promoting the formation of new blood vessels to support the burgeoning bone growth.
2. Osteoblast differentiation: BMP-2 exhibits impressive capabilities as an inducer of osteoblast differentiation. It sets in motion the differentiation process of mesenchymal stem cells into osteoblasts, which are responsible for bone formation and subsequent mineralization.
3. Bone regeneration: Notably, BMP-2 significantly contributes to bone regeneration by boosting the activity of osteoblasts and aiding in the formation of new bone tissue whose presence markedly stimulates bone regeneration processes to a pivotal aspect of overall bone health and healing (Figure 2).

SMADs represent pivotal regulatory proteins and downstream effectors within numerous signaling pathways. Among them, Smad1 and Smad5 are prominently associated with the BMP signaling pathway (57). Upon BMP activation of SMADs, these proteins augment their own gene expression by chromatin remodeling. Moreover, they recruit specific transcription factors, thereby regulating factors associated with bone development (58). Central to the regulation of osteoblast differentiation is Runx2, a critical transcription factor within the Runx family (59). Runx2 plays an essential role throughout all stages of mesenchymal stem cell differentiation into osteoblasts (60). Additionally, Runx2 regulates the expression of type I collagen alpha 1 (COL1A1) in osteoblasts and exerts influence over the proliferation of osteoprogenitor cells (59, 60).

PAP has shown the ability to activate essential signaling pathways, including BMP-2/Smad1 and Smad5/Runx2 (61). PAP’s activation leads to the upregulation of Smad1 and Smad5 expression, enhancement of the key transcription factor Runx2, elevation of bone-related ALP and OCN levels, and stimulation of the differentiation and mineralization of bone marrow mesenchymal stem cells into osteoblasts (62–64). This comprehensive action promotes bone formation, effectively addressing the problem of decreased bone mass seen in osteoporosis (61–64).

These findings underscore the potential of PAP in promoting bone regeneration and repair by modulating key signaling pathways involved in osteoblast differentiation and bone formation.

### MAPK/MMP-9 signaling pathway

The MAPK family encompasses four subtypes: extracellular signal-regulated kinase 1/2 (ERK1/2), p38, extracellular signal-regulated kinase 5 (ERK5), and c-Jun N-terminal kinase (JNK) (65). Notably, JNK and ERK1 are implicated in enhancing osteoclast differentiation and proliferation, ultimately amplifying bone resorption efficiency (66, 67).

Matrix metalloproteinases (MMPs) represent a vital protease family responsible for degrading the extracellular matrix, including collagen (68). This family comprises 23 different members, each characterized by distinct structural domains and functions (67). Specifically, MMP-9 demonstrates high expression and activity in osteoporotic bone tissue. Aside from its role in degrading the extracellular matrix, MMP-9 can regulate osteoclast gene expression, thereby compromising bone strength and resilience (69, 70).

PAP demonstrates the ability to diminish the activity of ERK1, JNK, and MMP-9 induced by retinoic acid in osteoporotic rats (71). Through this inhibition, PAP effectively reduces MMP-9-mediated degradation of the extracellular matrix and the consequent stimulation of osteoclasts (71). Additionally, PAP exerts inhibitory effects by reducing the release of osteocalcin (OCN) from bones into the blood, lowering the levels of tartrate-resistant acid phosphatase (TRACP) in the serum (71). These actions collectively counter bone resorption in individuals affected by osteoporosis.

Therefore, PAP may modulate the MAPK/MMP-9 signaling pathway, playing a role in maintaining bone mineral density and strength, ultimately contributing to maintaining a healthy bone structure and function.

### NF-κB signaling pathway

In fact, TNF-ɑ has multiple regulatory effects in bone metabolism. Not only does negatively impacts bone formation by osteoblasts but can also independently induce osteoclast differentiation with the involvement of p50, p52, and NF-κB (72, 73). NF-κB ligand coupling with RANKL leads to activation and differentiation of osteoclasts derived from mononuclear hematopoietic myeloid lineage cells, promoting bone resorption (74).

PAP can inhibit the NF-κB signaling pathway, downregulate the expression of p65 protein, improve osteoblast differentiation inhibited by TNF-ɑ, enhance the expression of the key transcription factor Runx2, increase the levels of osteoblast markers (such as OCN, ALP, OPN, BSP), promote osteoblast differentiation and mineralization, inhibit TNF-ɑ-induced osteoclast differentiation, resist bone resorption, and address the bone homeostasis imbalance in osteoporosis (39).

These findings highlight the potential therapeutic role of PAP in alleviating the detrimental effects of TNF-ɑ on bone health by modulating key signaling pathways to enhance osteoblast functionality and suppress osteoclast activity.

### EGF/EGFR signaling pathway

Epidermal Growth Factor (EGF) protein family is widely expressed in the skeletal system, prominently present in osteoblasts and osteoclasts (75). EGF, as a peptide composed of 53 amino acid residues, plays a crucial role in bone health (76, 77):

1. Promotion of osteoblast maturation: EGF and its receptor can increase the levels of osteocalcin (OCN) and alkaline phosphatase (ALP), both important markers of osteoblast activity, which promotes the maturation and functionality of osteoblasts.
2. Regulation of bone deposition: Ligands of the EGF receptor (EGFR) enhance bone deposition and bone matrix formation, achieving this by regulating the proliferation of osteoblasts and growing chondrocytes, which are critical for bone growth and modeling.

PAP has a significant anti-inflammatory and antioxidant effect through the EGF/EGFR signaling pathway, not only protecting osteoblasts and promoting bone formation but also inhibiting the induction of inflammatory and oxidative factors on osteoclasts, thereby reducing bone resorption (48):

1. Anti-inflammatory effect: PAP exhibits a notable anti-inflammatory effect by modulating the EGF/EGFR signaling pathway. This modulation leads to a reduction in the levels of pro-inflammatory cytokines in the serum, including TNF-ɑ, IL-1, and IL-6. By this mechanism, PAP effectively alleviates inflammation and helps in maintaining a balanced inflammatory response within the body.
2. Anti-oxidant effect: PAP demonstrates a remarkable ability to enhance the antioxidant defense mechanism. It achieves this by upregulating the expression of crucial factors such as nuclear factor erythroid 2-related factor 2 (Nrf2) and heme oxygenase-1 (HO-1). Additionally, PAP boosts the activity of superoxide dismutase (SOD), a vital antioxidant enzyme. Concurrently, it reduces the levels of malondialdehyde (MDA), a reliable marker of oxidative stress. These concerted actions contribute to PAP’s robust antioxidant properties, reinforcing the body’s defense against oxidative damage and stress.

In summary, EGF and its receptor play a significant role in bone biology. PAP utilizes the EGF/EGFR signaling pathway to exert anti-inflammatory and antioxidant effects, which can have a positive impact on bone homeostasis and overall health in osteoporosis.

### Insulin signaling pathway

Currently, there are four subtypes of insulin receptor substrates (IRS): IRS-1, IRS-2, IRS-3, and IRS-4 (78, 79). However, only IRS-1 and IRS-2 play crucial roles in regulating osteoblasts by the insulin signaling pathway in the growth and development of bones (78, 79). Insulin has been confirmed to induce osteoblast differentiation and proliferation, and regulate the synthesis of collagen and ALP through the ERK and PI3K pathways (80, 81).

PAP binds to IRS (insulin receptor substrate) and activates the insulin signaling pathway. This activation involves downstream insulin signaling molecules, including ERK and partial PI3K/Akt pathways. When osteoblasts co-cultured with PAP were exposed to ERK and PI3K inhibitors, it significantly reduced the mineralization of osteoblasts and ALP activity (82). Hence, PAP plays a crucial role in enhancing the vitality of osteoblasts and elevating the levels of ALP, OPN, and OCN through the insulin signaling pathway mediated by ERK and partial PI3K/Akt signaling (82). Ultimately, this modulation affects osteoblast differentiation, proliferation, maturation, and mineralization.

These discoveries underscore the vital involvement of the insulin signaling pathway in regulating osteoblast function and bone formation. By modulating this pathway, PAP demonstrates its potential to enhance bone formation in osteoporosis, presenting a promising avenue for therapeutic intervention.

## Conclusion and perspectives

In recent years, the aging of the global population has brought osteoporosis (OP) to the forefront as a critical global health concern. Due to the fact that OP has been steadily rising, the incidence of fractures imposes a significant burden on public health systems worldwide (83–85). It is imperative to comprehend the underlying mechanisms of OP to formulate effective strategies for its prevention and treatment. Bone homeostasis, characterized by a delicate balance between bone formation led by osteoblasts and bone resorption led by osteoclasts, is at the core of OP pathophysiology. However, the specific mechanisms that disrupt this balance have not been fully elucidated. Further research is essential to gain insights into the etiology, pathology, and physiology of OP. Exploring the intricate molecular and cellular processes that govern bone remodeling, the role of various signaling pathways, the impact of hormonal changes (such as those related to aging and hormone levels), and the contributions of genetic and environmental factors are all crucial areas of study in the quest to better understand and ultimately address OP.

PAP is a natural active ingredient extracted from the young antlers of Male Sika deer or red deer that have not yet ossified and are covered in hair. Research on PAP has been conducted in many Asian countries, including China, South Korea, and Japan, focusing on its molecular and cellular mechanisms. PAP has shown promise in regulating a variety of diseases, including those related to the cardiovascular system, skeletal system, and immune system (5, 86, 87).

In the context of bone metabolism, PAP has become a significant research focus, particularly in the field of osteoporosis. Studies have shown that PAP has various beneficial effects (36–49):

1. Regulating osteoblasts and promoting bone formation: PAP can regulate the proliferation, differentiation, maturation, and mineralization of osteoblasts, supporting the formation of new bone tissue.
2. Inhibiting osteoclast activity and resisting bone resorption: PAP can also inhibit the activity of osteoclasts by its anti-inflammatory and antioxidant propertiesn and increase the lifespan of osteoblasts as well as resist bone resorptionm, which helps maintain a healthy balance between bone formation and bone resorption.

As osteoporosis is characterized by imbalanced bone homeostasis with decreased bone formation and increased bone resorption, the ability of PAP to positively impact both osteoblasts and osteoclasts makes it a promising area of research in the field of osteoporosis, which offers potential as a therapeutic agent for improving bone health and addressing bone-related conditions.

PAP stands as a promising intervention to address compromised bone formation in osteoporosis. It achieves this by not only inhibiting bone resorption but also effectively maintaining bone homeostasis. PAP exerts its effects by multifaceted signaling pathways, including MAPK, EGF, NF-κB, BMP-2, insulin, ERK, and PI3K/Akt (38, 48, 61–64, 71, 82). These pathways collectively contribute to PAP’s capacity to enhance bone health and offer potential therapeutic benefits for individuals dealing with osteoporosis.

PAP does present certain limitations. Primarily, much of the research on PAP is derived from animal experiments, indicating a need for more extensive high-quality clinical randomized controlled trials specifically centered on PAP as the primary component in medications. Additionally, economically, the sourcing of deer antler, a key ingredient for PAP, can be relatively expensive, and the process to prepare PAP is associated with high costs. Pharmacologically, PAP faces challenges such as low bioavailability, a short half-life, and vulnerability to proteolytic degradation. However, comprehensive analysis regarding its pharmacokinetics and pharmacology is lacking, highlighting the need for further in-depth research and evaluation (5, 43). These limitations underscore the necessity for continued research and improvement in the development and application of PAP for potential clinical use.

Efforts are actively underway to enhance and advance PAP. Promising strategies to address its limitations are being explored. For instance, approaches such as utilizing PLGA microspheres or nano TCP/gelatin/PAP composite materials can significantly improve PAP’s bioavailability (43, 88). Moreover, optimizing the extraction process of PAP holds the potential to enhance its antioxidant capacity and overall safety (89). In terms of cost-effectiveness, techniques like ice acetic acid and ultrasonic fragmentation offer avenues to streamline production and reduce costs (90).

Considering these advancements and ongoing research endeavors, it is plausible to envision PAP and its derived products playing a pivotal role in the future management of osteoporosis. Continued research and innovative approaches will further shape the potential of PAP as a valuable component in the comprehensive management and treatment of osteoporosis.

## Author contributions

GW: Writing – original draft. YM: Writing – review & editing. WO: Writing – review & editing. CZ: Writing – review & editing, Conceptualization. WZ: Writing – review & editing, Conceptualization.

## Figures and Tables

**Table 1:** 

OP	Osteoporosis
OBs	Osteoblasts
OCs	Osteoclasts
PAP	Pilose antler polypeptide
Runx2	Runt-related transcription factor 2
ALP	Alkaline phosphatase
BSP	Bone sialoprotein
OCN	Osteocalcin
OPN	Osteopontin
BMP	Bone morphogenic proteins
Wnt	Wingless-related integration site
ERK	Extracellular signal regulated kinase
MAPK	Mitogen-activated protein kinase
JNK	C-Jun N-terminal kinase
MMP	Matrix metalloproteinase
RANKL	Receptor activator of NF-κB ligand
TNF	Tumor necrosis factor
IL	Interleukin
